# Long-term glycemic variability and risk of stroke in patients with diabetes: a meta-analysis

**DOI:** 10.1186/s13098-021-00770-0

**Published:** 2022-01-12

**Authors:** Xiaoli Ren, Zhiyun Wang, Congfang Guo

**Affiliations:** 1 Neurology Department, Tianjin First Central Hospital, 24 Fukang Road, Nankai District, Tianjin, 300192 China; 2 Health Management Center, Tianjin First Central Hospital, Tianjin, China

**Keywords:** Glycemic variability, HbA1c coefficient of variation, Glucose coefficient of variation, Stroke, Meta-analysis

## Abstract

**Objectives:**

Long-term glycemic variability has been related to increased risk of vascular complication in patients with diabetes. However, the association between parameters of long-term glycemic variability and risk of stroke remains not fully determined. We performed a meta-analysis to systematically evaluate the above association.

**Methods:**

Medline, Embase, and Web of Science databases were searched for longitudinal follow-up studies comparing the incidence of stroke in diabetic patients with higher or lower long-term glycemic variability. A random-effect model incorporating the potential heterogeneity among the included studies were used to pool the results.

**Results:**

Seven follow-up studies with 725,784 diabetic patients were included, and 98% of them were with type 2 diabetes mellitus (T2DM). The mean follow-up duration was 7.7 years. Pooled results showed that compared to those with lowest category of glycemic variability, diabetic patients with the highest patients had significantly increased risk of stroke, as evidenced by glycemic variability analyzed by fasting plasma glucose coefficient of variation (FPG-CV: risk ratio [RR] = 1.24, 95% confidence interval [CI] 1.11 to 1.39, P < 0.001; I^2^ = 53%), standard deviation of FPG (FPG-SD: RR = 1.16, 95% CI 1.02 to 1.31, P = 0.02; I^2^ = 74%), HbA1c coefficient of variation (HbA1c-CV: RR = 1.88, 95% CI 1.61 to 2.19 P < 0.001; I^2^ = 0%), and standard deviation of HbA1c (HbA1c-SD: RR = 1.73, 95% CI 1.49 to 2.00, P < 0.001; I^2^ = 0%).

**Conclusions:**

Long-term glycemic variability is associated with higher risk of stroke in T2DM patients.

## Background

People with diabetes are vulnerable to cardiovascular complications, which have become a major determinant for the prognosis of these patients [1, 2]. Among the cardiovascular complication of diabetes, stroke is a severe comorbidity which is associated with significant impaired quality of life and increased mortality of these populations [3–5]. Conventionally, persistent hyperglycemia evidenced by significantly increased plasma glucose or glycated hemoglobin (HbA1c) is well accepted as the major cause of vascular complications in patients with diabetes [6, 7]. However, subsequent studies showed that higher glycemic variability, which refers to increased fluctuation in glycemia, may also adversely affect the clinical outcomes in patients with diabetes [8–10]. Measuring glycemic fluctuation could be performed within or between days, which is named as short-term glycemic variability, or over weeks or months, which is named as long-term glycemic variability [11, 12]. Although no consensus has been reached regarding the standard definition or measuring methods for glycemic variability, coefficient of variation (CV) or standard deviation (SD) of visit-to-visit fasting plasma glucose (FPG) or HbA1c have been mostly applied for measuring of long-term glycemic variability in previous studies [13, 14]. Interestingly, previous studies evaluating the association between glycemic variability and stroke in people with diabetes showed inconsistent results [15–21]. Some studies showed that higher glycemic variability may be associated with higher risk of stroke [15, 17, 19, 21], while other studies did not [16, 18, 20]. Moreover, different parameters for long-term glycemic variability were applied among these studies, three with FPG-CV [15, 17, 19], two with FPG-SD [17, 19], four with HbA1c-CV [18–21], and the other four with [16, 18, 19, 21]. It remains unknown whether the difference in parameters used may affect the association between long-term glycemic variability and stroke. Besides, patients with diabetes may have multiple concurrent risk factors and comorbidities which may affect the risk of stroke in this population, such as aging, male sex, smoking, dyslipidemia, hypertension, and glycemic control status characterized by mean HbA1c etc. It is important to determine whether the possible association between long-term glycemic variability and stroke was independent of these risk factors. Therefore, we performed a meta-analysis to systematically evaluate the possible independent association between long-term glycemic variability measured by different metrics and the risk of stroke in people with diabetes.

## Methods

The meta-analysis was performed in accordance with the MOOSE (Meta-analysis of Observational Studies in Epidemiology) [22] and Cochrane’s Handbook [23] guidelines.

### Literature search

Studies were identified via systematic search of electronic databases of PubMed, Embase, and Web of Science via the following terms: (1) "glycemic" OR "glyceamic" OR "glucose" OR "hemoglobin A1c" OR "A1C" OR "HbA1c"; (2) "variability" OR "variation" OR "fluctuation"; and (3) "stroke" OR "transient ischemic stroke" OR "TIA" OR "cerebral infarction" OR "cerebrovascular infarction" OR "vascular" OR "cardiovascular". The search was limited to human studies published in English. The reference lists of related original and review articles were also analyzed using a manual approach. The final literature search was performed on June 20, 2021.

### Study selection

The inclusion criteria for the studies were: (1) the study design in longitudinal follow-up studies, including cohort studies, post-hoc analysis of clinical studies, and nested case–control studies; (2) included patients with confirmed diagnosis of diabetes, including type 1 and type 2 diabetes mellitus (T1DM and T2DM); (3) long-term glycemic variability was evaluated at baseline via visit-to-visit FPG or HbA1c, and quantified via the CV or SD of FPG or HbA1c; (4) evaluated the association between glycemic variability and incidence of stroke during follow-up; and (5) reported the risk ratio (RR) for the above association comparing patients with highest versus lowest category of glycemic variability in multivariate analyses. Reviews, editorials, cross-sectional studies, studies with non-diabetic patients, studies evaluating short-term glycemic variability, or studies irrelevant to the aim of current meta-analysis were excluded.

### Data extracting and quality evaluation

Literature search, data extraction, and quality assessment of the included studies were independently performed by two authors according to the predefined criteria. Discrepancies were resolved by consensus or discussion with the corresponding author. The extracted data included: (1) name of first author, publication year, and country where the study was performed; (2) study design characteristics; (3) patient characteristics, including diagnosis of the patients, sample size, mean age, and sex; (4) exposure characteristics, including parameters used for measuring of long-term glycemic variability at baseline, and definitions of highest and lowest glycemic variability among the included studies; (5) follow-up durations and outcomes reported; and (6) confounding factors that were adjusted. The quality of each study was evaluated using the Newcastle–Ottawa Scale [24] which ranges from 1 to 9 stars and judges each study regarding three aspects: selection of the study groups; the comparability of the groups; and the ascertainment of the outcome of interest.

### Statistical analyses

We used RRs and their corresponding 95% confidence intervals (CIs) as the general measure for association between long-term glycemic variability at baseline and incidence of stroke during follow-up. Data of RRs and their corresponding stand errors (SEs) were calculated from 95% CIs or P values, and were logarithmically transformed to stabilize variance and normalized the distribution [23]. The Cochrane’s Q test and estimation of I^2^ statistic were used to evaluate the heterogeneity among the include cohort studies [25]. A significant heterogeneity was considered if I^2^ > 50%. We used a random-effect model to synthesize the RR data because this model is considered as a more generalized method which incorporates the potential heterogeneity among the included studies [23]. Sensitivity analyses excluding studies with T1DM patients were performed. The potential publication bias was assessed by funnel plots with the Egger’s regression asymmetry test [26]. A P value < 0.05 indicates statistically significance. We used the RevMan (Version 5.1; Cochrane Collaboration, Oxford, UK) and Stata software for the meta-analysis and statistics.

## Results

### Literature search

**Fig. 1:**
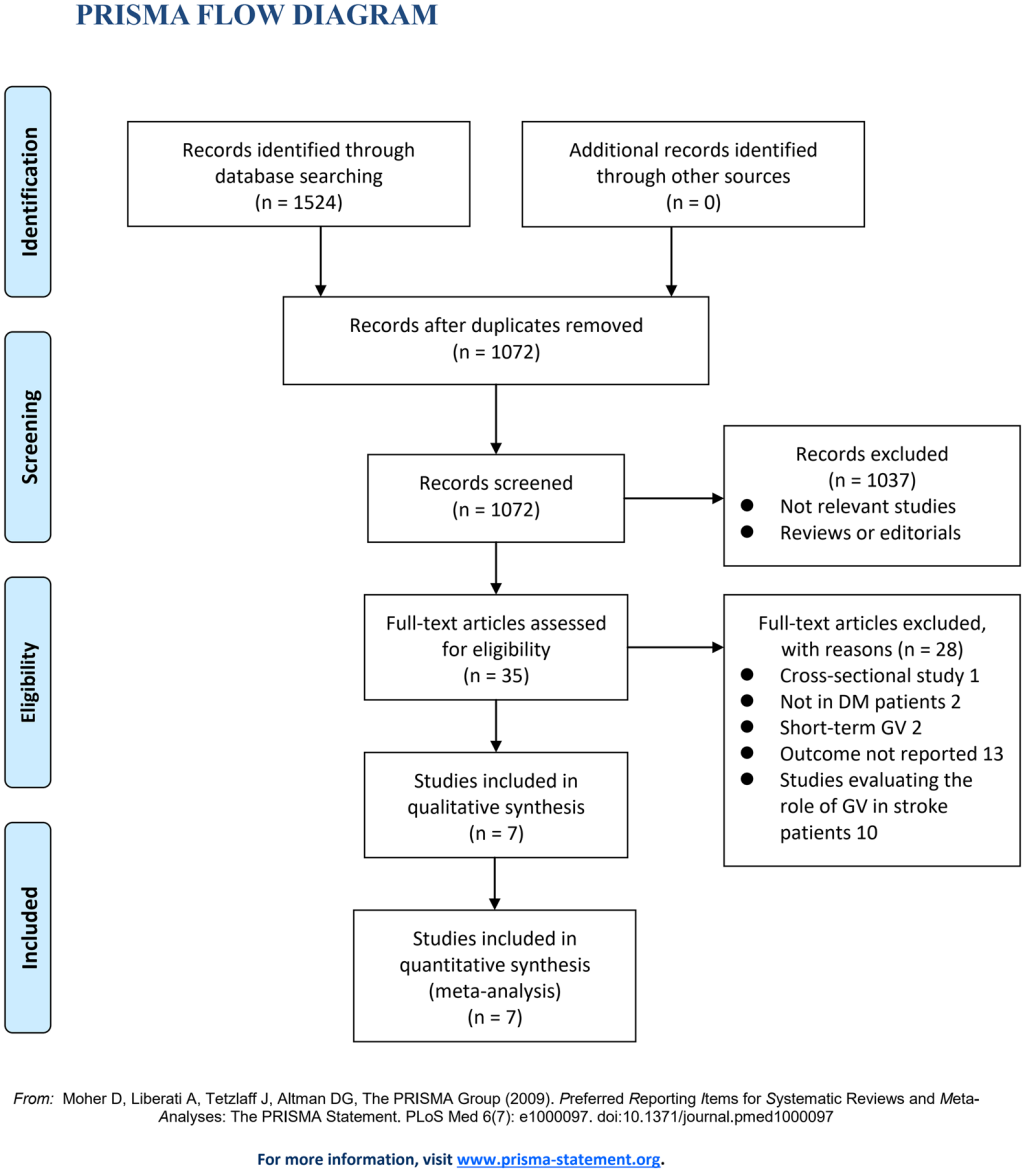
Flowchart of literature search

The process of database search was summarized in Fig. 1. Briefly, 1072 articles were found via initial literature search of PubMed, Embase, and Web of Science databases after excluding of the duplication, and 1037 were further excluded through screening of the titles and abstracts mainly because they were not relevant to the purpose of the meta-analysis. Subsequently, 35 potential relevant records underwent full-text review. Of these, 28 were further excluded based on reasons listed in Fig. 1. Finally, seven studies were included [15–21].

### Study characteristics and quality evaluation

The characteristics of the included studies were summarized in Table 1. Five of them were retrospective cohort studies [15–18, 21], and two of them were post-hoc analyses of clinical studies [19, 20]. These studies were published between 2014 and 2021, and performed in China, Japan, Korea, Scotland, Australia, and the United States, respectively. Six of the studies included T2DM patients, while the remaining one included primarily T2DM patients (97.5%) and a small proportion of T1DM patients (2.5%). In total, the meta-analysis included 725,784 diabetic, and 98% of them were with T2DM. At baseline, glycemic variability was measured with FPG-CV, FPG-SD, HbA1c-CV, and HbA1c-SD, and analyzed according to the tertiles, quartiles, and quintiles of the parameters. The calculation of FPG-CV, FPG-SD, HbA1c-CV, and HbA1c-SD were based on at least three measurements of FPG or HbA1c within first 1 to 2 years from baseline (Table 1). The mean follow-up duration was 7.7 years. As for the outcomes, incidence of ischemic stroke was reported in three studies [15, 16, 18], and the incidence of total stroke was reported in the other four studies [17, 19–21]. Possible confounding factors, such as age, sex, conventional cardiovascular risk factors, baseline glycemic status, other comorbidities, and concurrent antidiabetic treatments were adjusted among the included studies. The NOS scores of the included studies ranged from seven to eight, indicating generally good study quality (Table 2).

**Table 1:** Characteristics of the included stuides

Study	Country	Design	Participants	Sample size	Mean age Years	Male %	GV measurement and duration	GV parameter analysis	Follow-up duration Years	Outcome reported	Variables adjusted	Quality score
Lin (2014)	China	RC	T2DM patients	28,354	60.2	47.2	FPG-CV with first year	Q4:Q1	7.5	Ischemic stroke	Age, sex, obesity, smoking, alcohol, duration of DM, type OADs, hypertension drug treatment and HbA1c	8
Lee (2017)	China	RC	T2DM patients	8259	62	52	HbA1c-SD for at least 3 measurements	T3:T1	6.3	Ischemic stroke	Age, sex, hypertension, retinopathy and neuropathy, mean HbA1C, TG, HDL-c, eGFR, and medications use, including ACEI/ARB, aspirin, statin and/or fibrate, and insulin	8
Lee (2020)	Korea	RC	DM patients (T2DM 97.5%)	624,237	56.8	66.1	FPG-CV and FPG-SD for at least 3 measurements	Q4:Q1	8	Total stroke	Age, sex, BMI, alcohol drinking, smoking, regular exercise, presence of hypertension, dyslipidemia, CKD, lower income, duration of DM, OAD use, insulin use, and mean HbA1c	8
Li (2020)	Scotland	RC	T2DM patients	21,352	63.3	54.6	HbA1c-CV and HbA1c-SD for at least 5 measurements	Q5:Q1	6.8	Ischemic stroke	Age, sex, calendar year, Scottish Index of Multiple Deprivation quintiles, ever smoking, hypertension, BMI, HbA1c, HDL-c, eGFR, antiplatelet therapy at baseline, and CCI	8
Scott (2020)	Australia	Post-hoc	T2DM patients	9790	62.3	62.5	FPG-CV and FPG-SD, HbA1c-CV and HbA1c-SD for 3 measurements	Q4:Q1	5	Total stroke	Age, sex, HbA1c, study allocation, SBP, DM duration, prior CVD, prior microvascular complications and baseline use of OAD, insulin and antihypertensive drugs	7
Shen (2021)	USA	RC	T2DM patients	29,260	67.2	45.7	FPG-CV and FPG-SD for at least 4 measurements within first 2 years	Q4:Q1	4.2	Total stroke	Age, race, sex, smoking, BMI, SBP, non-HDL/HDL ratio, eGFR, HbA1c, insurance type, hypoglycemia events, use of OAD, anti-hypertensive medications, lipid-lowering medications, and antiplatelet and anticoagulant medications	7
Sato (2021)	Japan	Post-hoc	T2DM patients	4532	63	47.5	HbA1c-CV for at least 3 measurements	Q5:Q1	3.2	Total stroke	Age, sex, BMI, smoking, duration of DM, study allocation, hypertension, eGFR and HbA1c	7

*GV* glycemic variability, *RC* retrospective cohort, *T2DM* type 2 diabetes mellitus, *FPG* fasting plasma glucose, *HbA1c* hemoglobin A1c, *SD* standard deviation, *CV* coefficient of variation, *Q4: Q1* comparison between the fourth and the first quartiles, *T3: T1* comparison between the third and the first tertiles, *Q5: Q1* comparison between the fifth and the first quintiles, *DM* diabetes mellitus, *OAD* oral antidiabetic drug, *DM* diabetes mellitus, *BMI* body mass index, *TG* triglyceride, *HDL-c* high-density lipoprotein cholesterol, *HDL-c* low-density lipoprotein cholesterol, *CKD* chronic kidney disease, *eGFR* estimated glomerular filtrating rate, *SBP* systolic blood pressure, *CKD* chronic kidney disease, *CVD* cardiovascular disease, *ACEI* angiotensin converting enzyme inhibitor, *ARB* angiotensin II receptor blocker, *CCI* Charlson Comorbidity Index

**Table 2:** Details of study quality evaluation via the Newcastle–Ottawa Scale

Study	Representativeness of the exposed cohort	Selection of the non-exposed cohort	Ascertainment of exposure	Outcome not present at baseline	Control for age	Control for other confounding factors	Assessment of outcome	Enough long follow-up duration	Adequacy of follow-up of cohorts	Total
Lin (2014)	1	1	1	1	1	0	1	1	1	8
Lee (2017)	1	1	1	1	1	0	1	1	1	8
Lee (2020)	1	1	1	1	1	1	0	1	1	8
Li (2020)	1	1	1	1	1	0	1	1	1	8
Scott (2020)	0	1	1	1	1	1	0	1	1	7
Shen (2021)	1	1	1	1	1	0	0	1	1	7
Sato (2021)	0	1	1	1	1	0	1	1	1	7

### Long-term glycemic variability and stroke risk in people with diabetes

**Fig. 2:**
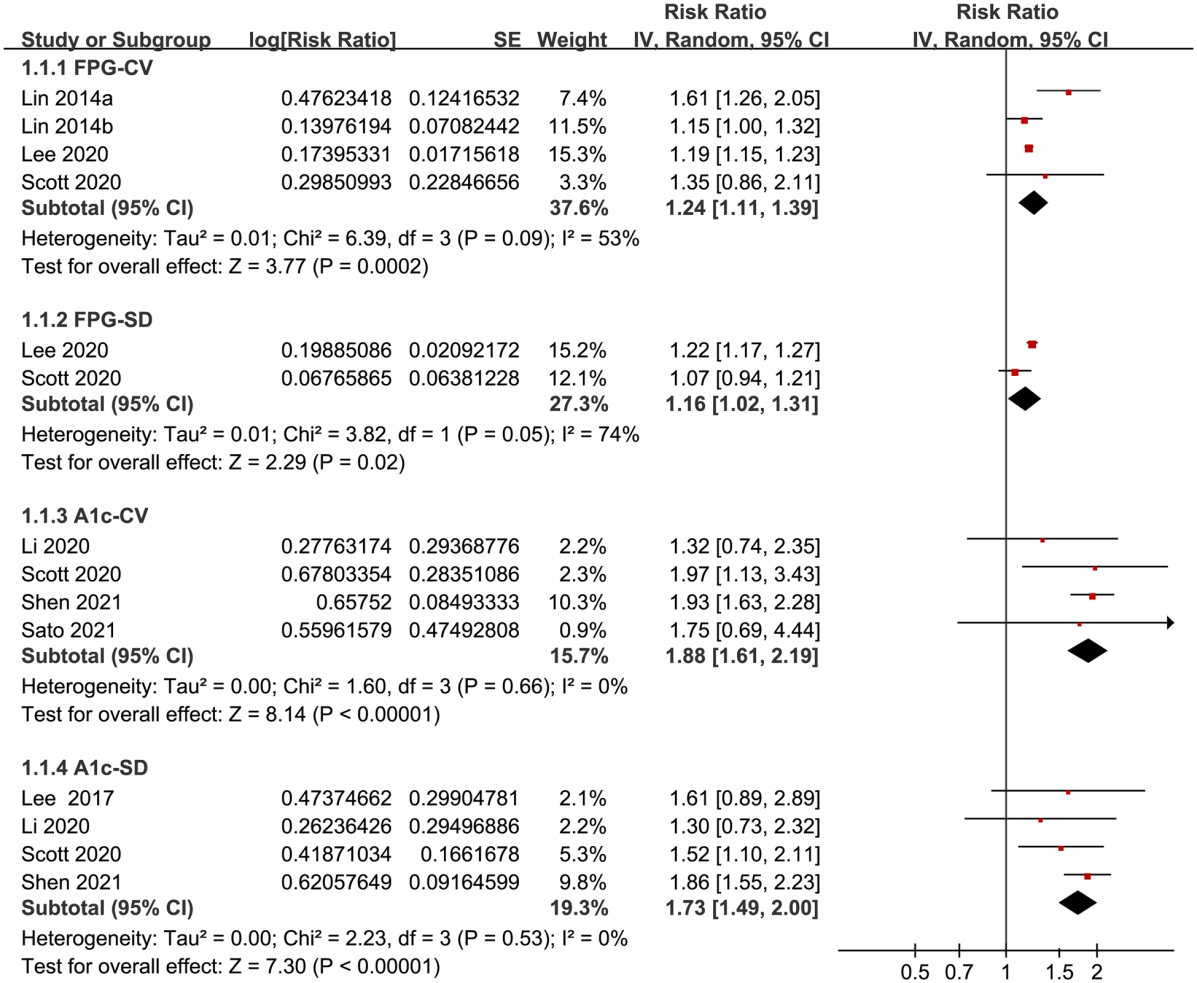
Forest plots for the meta-analysis of the association long-term glycemic variability and stroke risk in patients with diabetes. From upper to lower panels, long-term glycemic variability was measured by FPG-CV, FPG-SD, HbA1c-CV, and HbA1C-SD

Three studies [15, 17, 19] used FPG-CV to evaluate baseline glycemic variability. One [15] of the studies reported outcomes according to the baseline glycemic status of the patients (HbA1c < 7% or HbA1c ≥ 7%), these datasets were included in to the meta-analysis independently. Pooled results showed that higher FPG-CV was indepdently associated with higher risk of stroke in diabetic patients (highest versus lowest category of FPG-CV: RR = 1.24, 95% CI 1.11 to 1.39, P < 0.001; I^2^ = 53%; Fig. 2). Sensitivity analysis limited to studies with T2DM patients alone showed consistent results (RR: 1.33, 95% CI 1.05 to 1.70, P = 0.02; I^2^ = 65%). Meta-analysis with two studies [17, 19] also showed that higher FPG-SD was independently associated with higher risk of stroke (highest versus lowest category RR = 1.16, 95% CI 1.02 to 1.31, P = 0.02; I^2^ = 74%; Fig. 2). Sensitivity analysis was not performed since only two studies were included. Meta-analysis of four studies all with T2DM patients consistently showed that higher HbA1c-CV [18–21] and HbA1c-SD [16, 18, 19, 21] were both indepdently associated with higher risk of stroke (HbA1c-CV: RR = 1.88, 95% CI 1.61 to 2.19 P < 0.001; I^2^ = 0%; HbA1c-SD: RR = 1.73, 95% CI 1.49 to 2.00, P < 0.001; I^2^ = 0%; Fig. 2).

### Publication bias

**Fig. 3:**
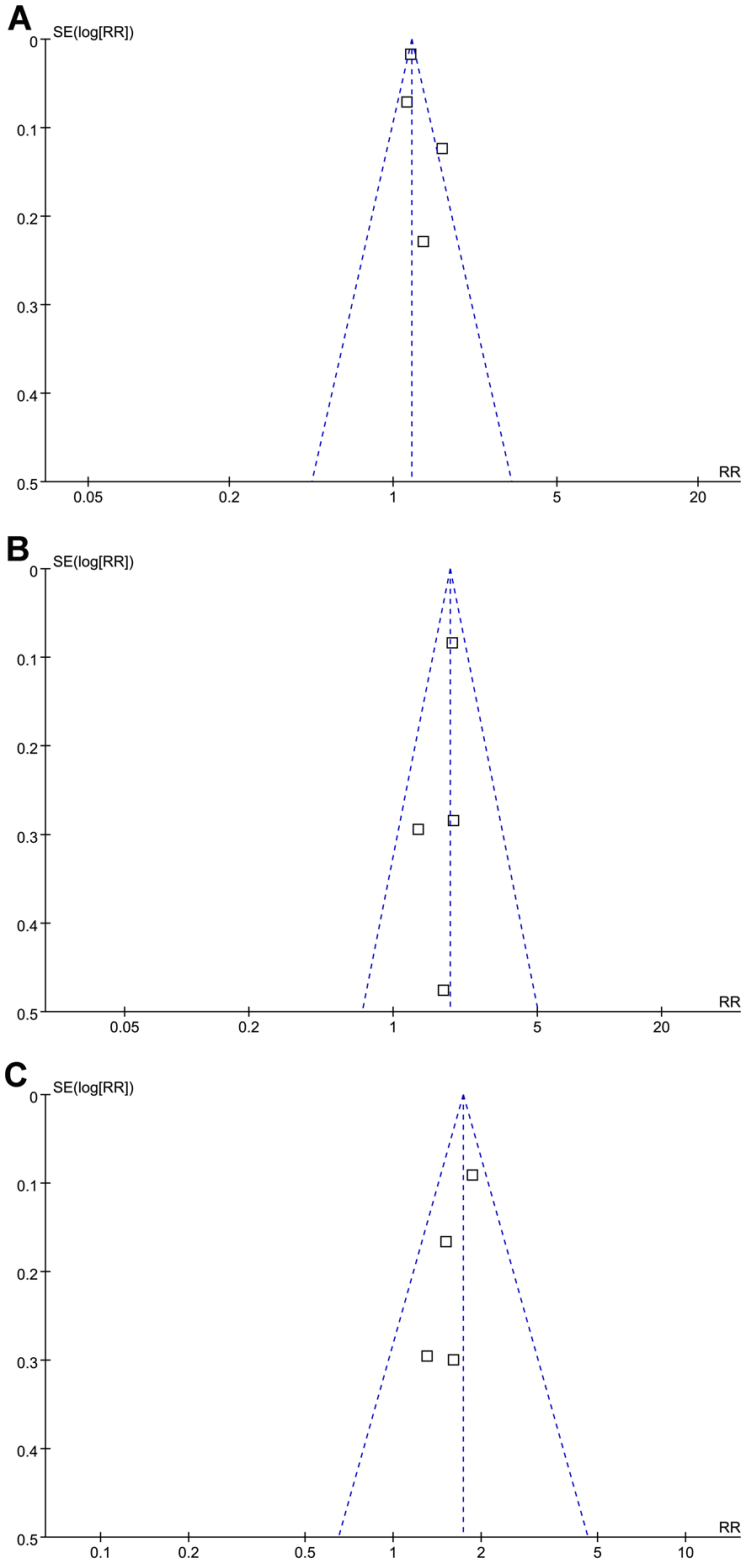
Funnel plots for the publication bias of the meta-analysis of the association long-term glycemic variability and stroke risk in patients with diabetes. **A** Funnel plots for studies analyzed with FPG-CV. **B** Funnel plots for studies analyzed with HbA1c-CV; and C, funnel plots for studies analyzed with HbA1C-SD

The funnel plots for the meta-analysis of the association between glycemic variability and stroke risk, as evaluated by FPG-CV, HbA1c-CV, and HbA1c-SD were shown in Fig. 3A–C. The plots were symmetrical on visual inspection, suggesting low risk of publication bias. Egger’s regression tests were not performed because only four datasets were available for each outcome. Publication bias for the association between FPG-SD and stroke risk could not be determined because only two studies were available for the outcome.

## Discussion

In this meta-analysis, by pooling the results of seven longitudinal follow-up studies, we found that increased long-term glycemic variability in patients with T2DM was significantly associated with higher risk of stroke, as evidenced by analyses with four commonly used metrics for long-term glycemic variability including FPG-CV, FPG-SD, HbA1c-CV, and HbA1c-SD. These results suggested that besides persistent hyperglycemia, increased long-term glycemic variability may also be an independent risk factor for stroke in T2DM patients.

To the best of our knowledge, this is the first meta-analysis that evaluated the association between long-term glycemic variability and risk of stroke in people with diabetes. A previous meta-analysis published in 2015 showed that increased glycemic variability was associated with increased risk of cardiovascular events in patients with T1DM and T2DM [10]. However, this meta-analysis pooled the results of studies with different metrics for long-term glycemic variability and more importantly, outcome of stroke incidence was not separately investigated [10]. In fact, this is not feasible because only one study [15] published before 2015 that assessed the association between long-term variability and incidence of stroke in T2DM patients with FPG-CV. Our study, instead, by pooling the results of updated longitudinal follow-up studies, showed that the four commonly used metrics for glycemic variability were all independently associated with the risk of stroke in patients with diabetes. Moreover, these results were based on longitudinal follow-up studies, which could therefore provide a temporal relationship between long-term glycemic variability and stroke. In addition, multivariate analyses were applied in all of the included studies, which may suggest that association between high glycemic fluctuation and stroke is independent of confounding factors such as conventional cardiovascular risk factors and average glycemic status indicated by HbA1c level.

The possible mechanisms underlying the association between glycemic fluctuation and stroke may be multifactorial. Pathophysiologically, glucose fluctuation, particularly the short-term glycemic fluctuation has been associated with the severity of systematic atherosclerosis, possibly via the inducing oxidative stress, inflammatory cytokines, and endothelial damage [27, 28]. Consistently, a recent cross-sectional study showed that glucose fluctuation is significantly associated with severe siphon stenosis of internal carotid artery in T2DM patients, suggesting that glucose fluctuation might be a risk factor for intracranial artery stenosis and ischemic stroke [29]. An early study also showed that time-scale-dependent glycemic fluctuations might contribute to brain atrophy and cognitive outcomes in patients with T2DM, which may deteriorate the cognitive function after stroke [30]. These studies suggested the adverse influence of increased glycemic variability on the pathogenesis and severity of stroke. Besides, it has also been hypothesized that additional mechanisms such as metabolic memory and insulin resistance may also be involved in the association between long-term glycemic variability and vascular complications of diabetes, including stroke [31, 32]. Using human umbilical vein endothelial cells (HUVECs) treated with oscillatory glucose to mimic the glycemic variability, a study showed that reactive oxygen species and vital markers of DNA damage were significantly elevated, which suggested the role of an underlying molecular mechanism contributing for the persistence of the damage leading to metabolic memory [33]. Future studies are warranted to elucidate the exact molecular pathways underlying the association between long-term glycemic variability and risk of stroke.

Interestingly, a recent clinical trial showed that optimized glucose management targeting glucose fluctuation can improve nerve function for patients with T2DM following the first ischemic stroke [34]. Although the findings of our meta-analysis should be validated in large-scale prospective studies, it could be hypothesized that reducing glucose fluctuation may further reduce the incidence of stroke in T2DM patients.

Our study has limitations. Firstly, studies available for the meta-analysis were retrospective, which may be confounded by the recall or selection biases. Therefore, prospective cohort studies are needed for validation. Secondly, limited datasets were available for each metrics of glycemic variability, and we were unable to evaluate the influences of study patient or study characteristics on the association, such as the age, sex, comorbidities, and follow-up durations. Besides, a dose–response relationship between long-term glycemic variability and stroke is important. However, we were unable to determine such relationship because these data were rarely reported among the included studies. Large-scale prospective studies are also warranted for further investigation. In addition, almost all studies included T2DM patients. Accordingly, the possible association between long-term glycemic variability and stroke risk in T1DM patients should be evaluated in future studies. Large-scale prospective studies with appropriate analyses are still needed to evaluate the association between long-term glycemic variability and risk for stroke in patients with T1DM and T2DM. Moreover, we were unable to determine whether the association was consistent for outcomes of ischemic and hemorrhagic stroke. Besides, although studies with multivariate analysis were included, we could not exclude the existence of residual factors that may affect the association, such as the concurrent use of antidiabetic medications that may reduce glycemic fluctuation. Finally, a causative relationship between high glycemic variability and stroke could not be derived based on our study, because it is a meta-analysis of observational studies. Clinical trials may be considered to evaluate whether reduce glycemic fluctuation could reduce the incidence of stroke in T2DM patients.

## Conclusions

In conclusion, results of the meta-analysis showed that increased long-term glycemic variability in patients with T2DM was significantly associated with higher risk of stroke. Glycemic fluctuation should be considered in the risk assessment for stroke and determination of optimized antidiabetic treatments in T2DM patients.

## Data Availability

The datasets generated and/or analyzed during the current study are available from the corresponding author on reasonable request.

## Abbreviations

SD: Standard deviation
FPG: Fasting plasma glucose
HbA1c: Glycated hemoglobin
MOOSE: Meta-analysis of Observational Studies in Epidemiology
T1DM: Type 1 diabetes mellitus
T2DM: Type 2 diabetes mellitus
